# Comparison between Whatman FTA Elute Cards and Conventional Swab for the Detection of Pathogenic Enteric Bacteria Using an RT-qPCR Assay

**DOI:** 10.1155/2021/9963047

**Published:** 2021-07-02

**Authors:** Na Yue, Zichao Jia

**Affiliations:** Department of Clinical Laboratory, Tianjin Medical University General Hospital, Tianjin 300070, China

## Abstract

The emergence of outbreaks of foodborne illness is closely associated with food contamination caused by various enteric pathogens, such as *Escherichia coli* O157:H7, *Listeria monocytogenes*, *Salmonella enterica* serovar Enteritidis, and *Staphylococcus aureus*. The control of enteric pathogens poses a challenge due to the fact that these pathogens can persist for a long period of time in the environment. The rapid detection of pathogenic organisms plays a crucial role in the prevention and identification of crises related to health, safety, and well-being. Improper sample handling and processing may influence the diagnostic efficacy and accuracy. The aim of the present study was to compare the preservation capacity for enteric bacteria between Whatman Flinders Technology Associates (FTA) cards and swabs for reverse transcription-quantitative PCR (RT-qPCR) detection. It was found that Whatman FTA cards exhibited an improved preservation capacity for five types (both laboratory and environmental strains) of enteric bacteria, including *Escherichia coli* O157:H7, *Listeria monocytogenes*, *Salmonella enterica* serovar Enteritidis, and *Staphylococcus aureus* for RT-qPCR detection. Hence, Whatman FTA cards may be a suitable tool for the routine isolation of foodborne bacteria for molecular diagnosis. Therefore, the use of Whatman FTA cards for sample collection and preservation may increase sensitivity and accuracy for bacteria isolation and diagnosis.

## 1. Introduction

The human intestinal microbiome plays an important role in maintaining health and preventing disease in the body [1]. The emergence of outbreaks of foodborne illness is closely related to food contamination caused by various enteric pathogens, which has been reported to cause high morbidity and mortality among humans worldwide [2]. The World Health Organization (WHO) reported that there were approximately a few million cases of foodborne illness caused by pathogenic bacteria each year [3]. The control of enteric pathogens poses a challenge, as these pathogens can persist for a long period of time in the environment [2].

There are few common enteric bacteria, including *Escherichia coli* O157:H7, *Listeria monocytogenes*, *Salmonella enterica* serovar Enteritidis, and *Staphylococcus aureus* [4]. These bacterial pathogens have been reported to often cause foodborne diseases and hospitalization among humans [5, 6]. *Escherichia coli* O157:H7 was first discovered in 1982 in Oregon and Michigan as a causative reagent for food contamination, which could cause serious illness, including abdominal pain, watery diarrhea, and hemorrhagic colitis [7]. As a foodborne pathogen, *Listeria monocytogenes* causes severe fatal gastrointestinal illness and is associated with a high mortality rate (i.e., 24%) [8]. *Salmonella enterica* serovar Enteritidis is an important Gram-negative intracellular pathogen with a broad host range, whose characteristic is that the pathogen can induce salmonellosis in humans and animals [9]. *Staphylococcus aureus* is a Gram-positive opportunistic pathogen, which is an important nosocomial pathogen [10]. *Staphylococcus aureus* has been reported to infect multiple tissues, including the intestine, skin, soft tissue, and lung [11]. The aforementioned four enteric pathogens pose a heavy burden to public health worldwide; thus, attention should be paid to their hazardous effects.

The rapid detection of pathogenic organisms plays a crucial role in prevention and identification of crises related to health, safety, and wellbeing [12]. It is challenging to diagnose pathogenic bacteria at very early stage due to the lack of sensitive detection methods [13]. Several diagnostic methods have been developed for the detection of bacteria, including PCR [14], filter paper method [15], sensor systems [16], and the Scan system method [17]. Among these methods, PCR is one of the most commonly used detection approaches, which was developed towards the end of the last century and is broadly used in the biology and medicine fields [17]. PCR is a relatively rapid detection method for bacterial identification, which normally requires between 5 and 24 h [18]. However, improper sample handling and processing may influence the diagnostic efficacy and accuracy [19]. Long-term storage and frozen samples may decrease the number of bacteria, which can lead to false-negative results [20]. Therefore, it is crucial to select an optimal handling method for the detection of bacteria when using PCR assay.

Flinders Technology Associates (FTA) cards were specifically developed to simplify the handling and processing of nucleic acids [21]. An FTA card is comprised of a cotton-based cellulose paper treated with anionic detergents and buffer, which provides a stable matrix for the immobilization of genomes for molecular characterization, but is free from the living host cells or organism [22]. The use of FTA cards for the processing of samples for pathogen identification has been reported [23]. For PCR, a previous study has found that FTA cards can be used to diagnose a number of types of pathogen, including HIV, pathogenic fungi, etc. [24]. However, information regarding the effects of FTA cards on PCR detection is still limited [25]. The present study compared the differences between Whatman™ FTA Elute cards and conventional swabs for the handling of enteric bacteria, including *Escherichia coli* O157:H7, *Listeria monocytogenes*, *Salmonella enterica* serovar Enteritidis, and *Staphylococcus aureus* when detected by reverse transcription-quantitative PCR (RT-qPCR) assay. The information presented herein may be helpful for selecting the optimal handling and process method for bacterial diagnosis.

## 2. Materials and Methods

### 2.1. Reagents

BeyoPure™ LB Broth (premixed powder, cat. no. ST156, Beyotime Institute of Biotechnology) and BeyoPure™ LB Broth with Agar (premixed powder) (cat. no. ST158) were purchased from Beyotime Institute of Biotechnology. Whatman FTA Elute cards were obtained from Whatman plc; Cytiva (Whatman WB120315).

### 2.2. Bacteria

*Escherichia coli* O157:H7, *Listeria monocytogenes*, *Salmonella enterica* serovar Enteritidis, and *Staphylococcus aureus* were obtained from the Tianjin Medical University General Hospital Bacteria Bank. *Escherichia coli* O157:H7 was cultured in Luria-Bertani (LB) medium at 37°C with shaking at 180 rpm for 18–24 h. For enumeration, the culture was serially diluted in PBS and plated on LB agar incubated at 37°C for a further 22–24 h. Based on the counting results, the sample was serially diluted with deionized water to seven various concentrations (1 × 10^7^ CFU/ml, 5 × 10^6^ CFU/ml, 2.5 × 10^5^ CFU/ml, 6.25 × 10^4^ CFU/ml, 3.125 × 10^4^ CFU/ml, 3.125 × 10^4^ CFU/ml, and 7.8125 × 10^3^ CFU/ml). Various concentrations of bacteria were dropped on a Whatman FTA card and swab and stored at 4°C for 2 days for use in further experiments.

For *Listeria monocytogenes*, seed stocks of the bacteria were stored in *Listeria* enrichment broth (LEB) and 16.7% glycerol at −80°C. To grow the bacteria, 10 ml of sterile LEB was aliquoted into a culture tube, inoculated, and placed in a shaker/incubator overnight at 250 rpm (37°C). The number of bacteria was counted, and was subsequently serially diluted with deionized water to seven various concentrations (1 × 10^7^ CFU/ml, 5 × 10^6^ CFU/ml, 2.5 × 10^5^ CFU/ml, 6.25 × 10^4^ CFU/ml, 3.125 × 10^4^ CFU/ml, 3.125 × 10^4^ CFU/ml, and 7.8125 × 10^3^ CFU/ml). Various concentrations of bacteria were dropped on a Whatman FTA card and swab and stored at 4°C for 2 days for use in further experiments.

For *Salmonella enterica* serovar Enteritidis, one single typical colony was inoculated in 10 ml sterile brain heart infusion (BHI) broth, vortexed, and incubated at 37°C overnight (∼16 h), followed by counting the number of bacteria, and was subsequently serially diluted with deionized water to seven various concentrations (1 × 10^7^ CFU/ml, 5 × 10^6^ CFU/ml, 2.5 × 10^5^ CFU/ml, 6.25 × 10^4^ CFU/ml, 3.125 × 10^4^ CFU/ml, 3.125 × 10^4^ CFU/ml, and 7.8125 × 10^3^ CFU/ml).

In terms of *Staphylococcus aureus*, a single colony of bacteria was incubated in 10 ml Tryptone Soy Broth with 0.3% Yeast Extract (TSB-YE) at 37°C overnight with shaking at 190 rpm, followed by counting the number of bacteria, and was subsequently serially diluted with deionized water to seven various concentrations (1 × 10^7^ CFU/ml, 5 × 10^6^ CFU/ml, 2.5 × 10^5^ CFU/ml, 6.25 × 10^4^ CFU/ml, 3.125 × 10^4^ CFU/ml, 3.125 × 10^4^ CFU/ml, and 7.8125 × 10^3^ CFU/ml). All bacterial samples were stored at 4°C for use in following experiments.

### 2.3. Food Sample Sampling Process Using Whatman FTA Elute Cards and Swabs

Food samples containing *Escherichia coli* O157:H7, *Listeria monocytogenes*, *Salmonella enterica* serovar Enteritidis, and *Staphylococcus aureus* were obtained from the bio-sample bank of Tianjin Medical University General Hospital. Pathogens in food samples were diagnosed prior to storage in the bio-sample bank. Food samples were cut into sections, followed by being diluted with 1 ml sterile water, and filtered. The filtrate was dropped on Whatman FTA cards and swabs and stored at 4°C for 2 days for further experiments, respectively.

### 2.4. DNA Template Preparation

Crude DNA was isolated from bacterial samples on the FTA cards and swabs. DNA isolation was performed using a Genomic DNA Mini Preparation kit with Spin Colum (cat. no. D0063Beyotime Institute of Biotechnology) according to the manufacturer's instructions. Briefly, bacteria on the FTA cards and swabs were eluted with 180 *μ*l lysis buffer A, followed by the addition of 20 *μ*l proteinase K, and incubation in a water bath (37°C) for 10 min. Subsequently, the samples were supplemented with 200 *μ*l lysis buffer B, followed by the addition of 200 *μ*l ethanol, and were completely mixed. The samples were then added to the DNA purification spin column, followed by spinning down at >6,000 × g. Subsequently, spin columns with DNA were washed with wash buffer I and II, followed by spinning down at >18,000 × g to dry the filter of the spin column. DNA was eluted with 50 *μ*l elution buffer for use in further experiments.

### 2.5. RT-qPCR

RT-qPCR was performed using the iCycler iQ real-time detection system (Bio-Rad Laboratories, Inc.) according to manufacturer's instructions. Briefly, a bacterium DNA (2 *μ*l) was added to a hot-start reaction mixture containing SYBR-Green mix (BeyoFast™ SYBR-Green qPCR Mix; cat. no. D7260, Beyotime Institute of Biotechnology) and primers (Table 1). The program of RT-qPCR included an initial step of 95°C for 10 min followed by an amplification program for 40 cycles of 3 sec at 95°C, 5 sec at 61°C, and 20 sec at 72°C with fluorescence acquisition at the end of each extension. Subsequently, a melt program consists of 60 sec at 95°C, 60 sec at 65°C, and a gradual increase to 90°C at a rate of 0.2°C/sec with fluorescence acquisition at each temperature transition. The genes, including stx1 and stx2, were measured for *Escherichia coli* O157:H7, as that these two genes are two major virulence factors and often used for the diagnosis of *Escherichia coli* O157:H7 [26]. HlyA164 and hlyA177 are commonly used for the diagnosis of *Listeria monocytogenes* [27]. InvA, as one of the major virulence genes of *Salmonella enterica* serovar Enteritidis, is often used for the detection of this bacteria [28]. FemB is one of the genes encoding proteins which influences the level of methicillin resistance of *Staphylococcus aureus*, and it is often used for the detection of *Staphylococcus aureus* [29].

### 2.6. Statistical Analysis

The data were processed using GraphPad Prism (v8.0.2.263). One-way ANOVA followed by the Bonferroni test was applied to verify significant differences between groups. Differences with *P* < 0.05 were considered to indicate a statistically significant difference.

## 3. Results

### 3.1. CT Value of *Escherichia coli* O157:H7 Genes in Whatman FTA Elute Cards Is Higher than in Swabs

**Figure 1:**
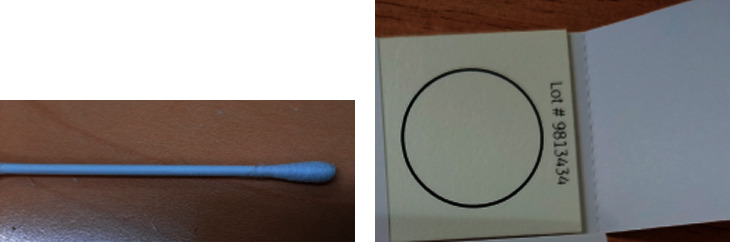
Conventional swab and Whatman FTA card.

**Figure 2:**
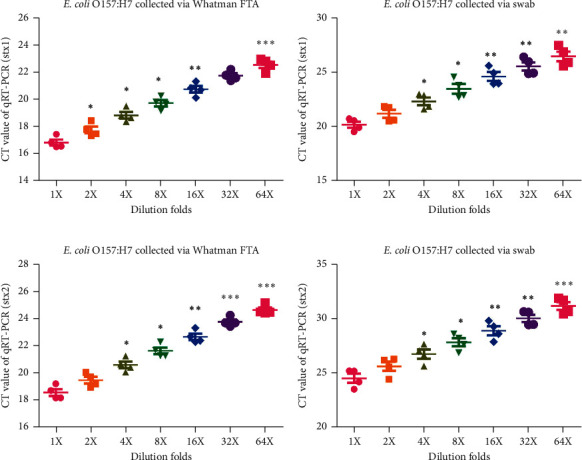
CT values of *Escherichia coli* O157:H7 genes in samples on Whatman FTA elute cards are higher than those on swabs. (a) Expression level of the stx1 gene in a serially diluted laboratory strain of *Escherichia coli* O157:H7 samples on Whatman FTA cards detected by RT-qPCR (*n* = 6, ^*∗*^*P* < 0.05, ^∗∗^*P* < 0.01, and ^∗∗∗^*P* < 0.001). (b) Expression level of the stx1 gene in a serially diluted laboratory strain of *Escherichia coli* O157:H7 samples on swabs detected by RT-qPCR (*n* = 6, ^*∗*^*P* < 0.05 and ^∗∗^*P* < 0.01). (c) Expression level of the stx2 gene in a serially diluted laboratory strain of *Escherichia coli* O157:H7 samples on Whatman FTA cards detected by RT-qPCR (*n* = 6, ^*∗*^*P* < 0.05, ^∗∗^*P* < 0.01, and ^∗∗∗^*P* < 0.001). (d) Expression level of the stx2 gene in a serially diluted laboratory strain of *Escherichia coli* O157:H7 samples on swabs detected by RT-qPCR (*n* = 6, ^*∗*^*P* < 0.05, ^∗∗^*P* < 0.01, and ^∗∗∗^*P* < 0.001). RT-qPCR: reverse transcription-quantitative PCR; FTA: Flinders Technology Associates.

To compare the ability to preserve bacterial samples between Whatman Elute FTA cards and conventional swabs (Figure 1), 200 *μ*l *Escherichia coli* O157:H7 was added on a Whatman Elute FTA card and conventional swab, followed by the detection of the expression of the stx1 and stx2 genes. It was found that the CT values of the stx1 gene of *Escherichia coli* O157:H7 on the Whatman Elute FTA card were significantly lower than those on the conventional swab (Figures 2(a) and 2(b), *n* = 6; *P* < 0.05, *P* < 0.01, and *P* < 0.001). Similarly, it was found that the CT values of the stx2 gene of *Escherichia coli* O157:H7 on the Whatman Elute FTA card were significantly lower than those on the conventional swab (Figures 2(c) and 2(d), *n* = 6; *P* < 0.05, *P* < 0.01, and *P* < 0.001). Thus, it was confirmed that the preservation capacity to *Escherichia coli* O157:H7 of the Whatman Elute FTA card was better than that of the conventional swabs.

**Figure 3:**
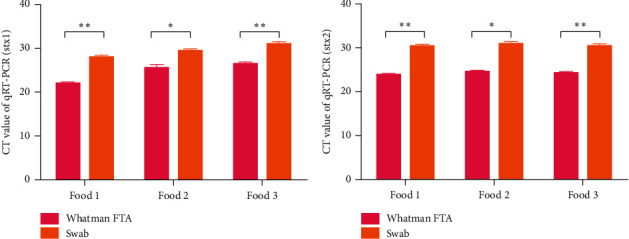
Detection of *Escherichia coli* O157:H7 genes in food samples containing *Escherichia coli* O157:H7 contamination by RT-qPCR collected using Whatman FTA cards and swabs. (a) Expression level of the stx1 gene in a serially diluted environmental strain of *Escherichia coli* O157:H7 samples on Whatman FTA cards and swabs detected by RT-qPCR (*n* = 6, ^*∗*^*P* < 0.05 and ^∗∗^*P* < 0.01). (b) Expression level of the stx2 gene in an environmental strain of *Escherichia coli* O157:H7 samples on Whatman FTA cards and swabs detected by RT-qPCR (*n* = 6, ^∗∗^*P* < 0.01). RT-qPCR: reverse transcription-quantitative PCR; FTA: Flinders Technology Associates.

To further determine the difference in the capacity for bacterial preservation between the Whatman Elute FTA card and conventional swabs, food samples containing *Escherichia coli* O157:H7 were collected with the Whatman Elute FTA card and conventional swabs. After 48 h, the expression of stx1 and stx2 was examined by RT-qPCR. It was found that the CT values of the stx1 and stx2 gene of *Escherichia coli* O157:H7 on the Whatman Elute FTA card were significantly lower than those on the conventional swab for three food samples (Figures 3(a) and 3(b), *n* = 6; *P* < 0.05 and *P* < 0.01).

### 3.2. CT Values of *Listeria monocytogenes* Genes in Whatman FTA Elute Cards Are Higher than Those in Swabs

**Figure 4:**
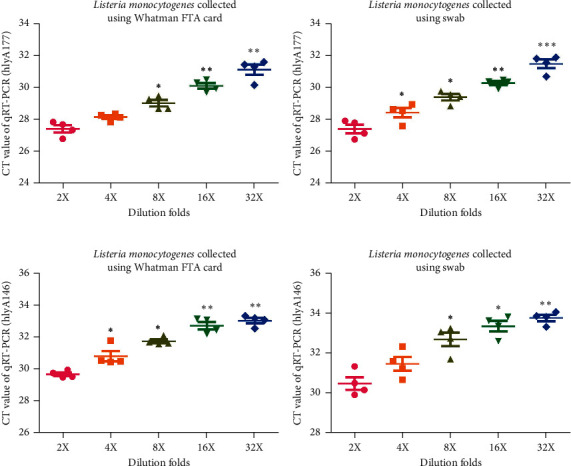
CT values of *Listeria monocytogenes* genes in samples on Whatman FTA Elute cards are higher than those on swabs. (a) Expression level of the hly177 gene in a serially diluted laboratory strain of *Listeria monocytogenes* samples on Whatman FTA cards detected by RT-qPCR (*n* = 6, ^*∗*^*P* < 0.05 and ^∗∗^*P* < 0.01). (b) Expression level of the hly177 gene in a serially diluted laboratory strain of *Listeria monocytogenes* samples on swabs detected by RT-qPCR (*n* = 6, ^*∗*^*P* < 0.05 and ^∗∗^*P* < 0.01). (c) Expression level of the hly146 gene in a serially diluted laboratory strain of *Listeria monocytogenes* samples on Whatman FTA cards detected by RT-qPCR (*n* = 6, ^*∗*^*P* < 0.05, ^∗∗^*P* < 0.01, and ^∗∗∗^*P* < 0.001). (d) Expression level of the hly146 gene in a serially diluted laboratory strain of *Listeria monocytogenes* samples on swabs detected by RT-qPCR (*n* = 6, ^*∗*^*P* < 0.05, ^∗∗^*P* < 0.01, and ^∗∗∗^*P* < 0.001). RT-qPCR: reverse transcription-quantitative PCR; FTA: Flinders Technology Associates.

*Listeria monocytogenes* is one of main foodborne bacteria; therefore, the difference in the preservation ability of Whatman FTA Elute cards and swabs for *Listeria monocytogenes* was compared by RT-qPCR assay. It was found that the expression of the *Listeria monocytogenes* gene, hlyA177, was significantly higher in samples collected using Whatman FTA Elute cards than those collected with swabs (Figures 4(a) and 4(b), *n* = 5; *P* < 0.05, *P* < 0.01, and *P* < 0.001). Another gene named hlyA164 of *Listeria monocytogenes* was also detected by RT-qPCR, which indicated that its expression was significantly higher in samples collected using Whatman FTA Elute cards than those collected using swabs (Figures 4(c) and 4(d), *n* = 5; *P* < 0.05 and *P* < 0.01).

**Figure 5:**
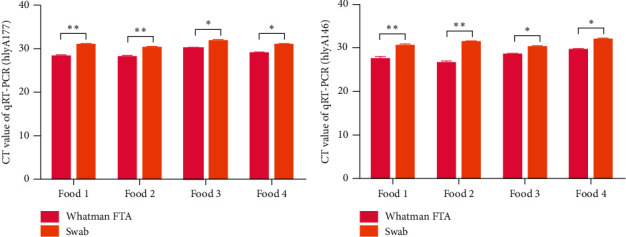
Detection of *Listeria monocytogenes* genes in food samples containing *Listeria monocytogenes* contamination by RT-qPCR collected using Whatman FTA cards and swabs. (a) Expression level of the hly177 gene in a serially diluted environmental strain of *Listeria monocytogenes* samples on Whatman FTA cards and swabs and detected by RT-qPCR (*n* = 6, ^*∗*^*P* < 0.05 and ^∗∗^*P* < 0.01). (b) Expression level of the hly146 gene in an environmental strain of *Listeria monocytogenes* samples on Whatman FTA cards and swabs detected by qRT-PCR (*n* = 6, ^*∗*^*P* < 0.05 and ^∗∗^*P* < 0.01). RT-qPCR: reverse transcription-quantitative PCR; FTA: Flinders Technology Associates.

To further investigate the difference in the capacity for bacterial preservation for *Listeria monocytogenes* between the Whatman Elute FTA cards and conventional swabs, four food samples containing *Listeria monocytogenes* contamination were collected using Whatman Elute FTA cards and conventional swabs. After 48 h, the expression of stx1 and stx2 was examined by RT-qPCR. It was indicated that the CT values of the hlyA177 and hlyA164 genes of *Listeria monocytogenes* on the Whatman Elute FTA card were significantly lower than those on the conventional swab for three food samples (Figures 5(a) and 5(b), *n* = 6; *P* < 0.05 and *P* < 0.01).

### 3.3. CT Values of the *Salmonella enterica* Serovar Enteritidis Gene in Whatman FTA Elute Cards Are Higher than Those on Swabs

**Figure 6:**
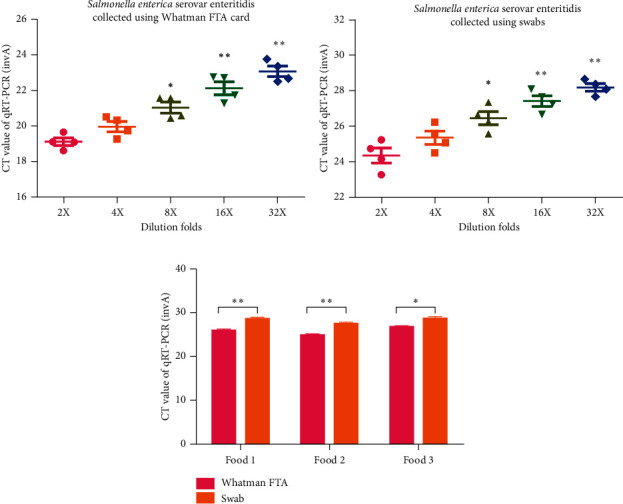
CT values of the *Salmonella enterica* serovar Enteritidis gene in Whatman FTA Elute cards are higher than those on swabs. (a) Expression of the *Salmonella enterica* serovar Enteritidis gene invA in samples collected using Whatman FTA Elute cards detected by RT-qPCR (*n* = 6, ^*∗*^*P* < 0.05 and ^∗∗^*P* < 0.01). (b) Expression of the *Salmonella enterica* serovar Enteritidis gene invA in samples collected using swabs detected by RT-qPCR (*n* = 6, ^*∗*^*P* < 0.05 and ^∗∗^*P* < 0.01). (c) Expression level of the invA gene in an environmental strain of *Salmonella enterica* serovar Enteritidis samples on Whatman FTA cards and swabs detected by RT-qPCR (*n* = 6, ^*∗*^*P* < 0.05 and ^∗∗^*P* < 0.01). RT-qPCR: reverse transcription-quantitative PCR; FTA: Flinders Technology Associates.

*Salmonella enterica* serovar Enteritidis is another main foodborne pathogen. Thus, the difference in the preservation ability for *Salmonella enterica* serovar Enteritidis of Whatman FTA Elute cards and swabs was compared by RT-qPCR assay. It was found that the expression of the *Salmonella enterica* serovar Enteritidis gene, invA, was significantly lower in samples collected using Whatman FTA Elute cards than in those collected using swabs (Figures 6(a) and 6(b), *n* = 5; *P* < 0.05 and *P* < 0.01). To further examine the difference in the capacity for bacterial preservation for *Salmonella enterica* serovar Enteritidis between Whatman Elute FTA card and conventional swabs, three food samples containing *Salmonella enterica* serovar Enteritidis contamination were collected using Whatman Elute FTA cards and conventional swabs. After 48 h, the expression of the invA was examined by RT-qPCR. It was indicated that the CT values of invA of *Salmonella enterica* serovar Enteritidis on Whatman Elute FTA cards were significantly lower than those on conventional swabs for the three food samples (Figure 6(c), *n* = 6; *P* < 0.05 and *P* < 0.01).

### 3.4. CT Values of the *Staphylococcus aureus* Gene in Whatman FTA Elute Cards Are Higher than Those in Swabs

**Figure 7:**
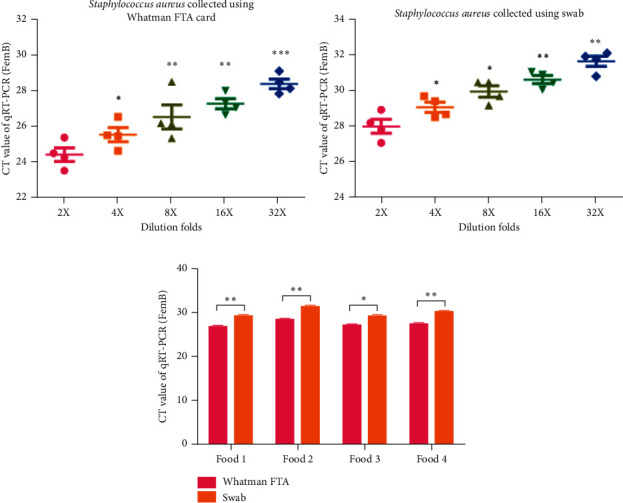
CT values of the *Staphylococcus aureus* gene in Whatman FTA Elute cards are higher than those on swabs. (a) Expression of the *Staphylococcus aureus* gene FemB in samples collected using Whatman FTA Elute cards detected by RT-qPCR (*n* = 6, ^*∗*^*P* < 0.05, ^∗∗^*P* < 0.01, and ^∗∗∗^*P* < 0.001). (b) Expression of the *Staphylococcus aureus* gene FemB in samples collected using swabs detected by RT-qPCR (*n* = 6, ^*∗*^*P* < 0.05 and ^∗∗^*P* < 0.01). (c) Expression level of the FemB gene in an environmental strain of *Staphylococcus aureus* samples on Whatman FTA cards and swabs detected by RT-qPCR (*n* = 6, ^*∗*^*P* < 0.05 and ^∗∗^*P* < 0.01). RT-qPCR: reverse transcription-quantitative PCR; FTA: Flinders Technology Associates.

*Staphylococcus aureus* can cause severe food poisoning, and the diagnosis of the pathogen remains challenging in food or the environment [25]. Thus, the present study compared the difference in the preservation ability for *Staphylococcus aureus* of Whatman FTA Elute cards and swabs by RT-qPCR assay. It was found that expression of the *Staphylococcus aureus* gene, FemB, was significantly higher in samples collected using Whatman FTA Elute cards than those collected using swabs (Figures 7(a) and 7(b), *n* = 6; *P* < 0.05, *P* < 0.01, and *P* < 0.001). To further examine the difference in the capacity for bacterial preservation for *Staphylococcus aureus* between Whatman Elute FTA cards and conventional swabs, three food samples containing *Staphylococcus aureus* contamination were collected using Whatman Elute FTA cards and conventional swabs. After 48 h, the expression of the FemB gene was examined bu RT-qPCR. It was indicated that the CT values of FemB of *Staphylococcus aureus* on Whatman Elute FTA cards were significantly lower than those on conventional swabs for the three food samples (Figure 7(c), *n* = 6; *P* < 0.05 and *P* < 0.01).

## 4. Discussion

Foodborne disease outbreak remains a major challenge for public health worldwide [30]. Enteric bacteria, such as *Escherichia coli* O157:H7, *Listeria monocytogenes*, *Salmonella enterica* serovar Enteritidis, and *Staphylococcus aureus*, are considered as main pathogens which cause foodborne disease [7]. Accurate and rapid diagnosis plays a crucial in the prevention of infections from these enteric pathogens. The present study compared the differences in the preservation ability of Whatman FTA Elute cards and swabs for *Escherichia coli* O157:H7, *Listeria monocytogenes*, *Salmonella enterica* serovar Enteritidis, and *Staphylococcus aureus* by RT-qPCR assay. It was found that Whatman FTA Elute cards had a stronger preservation capacity for these five enteric bacteria compared with conventional swabs. Thus, it was confirmed that Whatman FTA Elute cards may be a suitable tool for collecting samples of *Escherichia coli* O157:H7, *Listeria monocytogenes*, *Salmonella enterica* serovar Enteritidis, and *Staphylococcus aureus* when diagnosis is made by RT-qPCR assay. The information presented herein may prove to be useful for the diagnosis and prevention of infections from enteric bacteria.

Collection, sample storage, and bacterial DNA extraction methods play an important role in accurately detecting intestinal bacteria [31]. Swab collection is one of the most commonly used methods for the isolation and/or detection of bacteria using molecular methods [32]. However, the storage temperature and storage duration will influence the isolation and molecular detection of bacteria [33]. Although the swab collection method has been used for viral or bacterial collection for decades, some disadvantages have been reported including the following: (i) the swab market can be disorderly and includes a large number of manufacturers without production licenses; (ii) swabs containing fresh samples of highly pathogenic microorganisms still have infection risks; (iii) swabs are easily contaminated. Therefore, an improved sample collection method is required compared with swabs for pathogenic bacterial isolation for detection by the molecular method.

FTA matrix cards developed by Whatman have been demonstrated to be suitable for the rapid collection, purification, and analysis of genetic material from a wide range of biological sources, such as whole blood, buccal scrapes, tissues, plasmids, plant material, and microorganisms [34]. Whatman FTA cards have been used for the isolation of a variety of pathogens, including viruses, bacteria, and fungus for molecular detection [35]. Several studies have confirmed the advantages of Whatman FTA cards in collecting pathogenic samples. In the study performed by Sierra-Arguello et al. [34], it was found that 100 bacteria samples were successfully amplified using the 16S rDNA gene and demonstrated by DNA sequencing following storage for 3 years at an ambient temperature, which manifested the robust preservation capacity for bacteria of FTA cards. Fowler et al. [36] made use of Whatman FTA cards to store DNA for a long period of time, followed by analyzing single nucleotide polymorphism (SNP); they found that DNA could be isolated from all samples of Whatman FTA cards, which indicated the potent ability of FTA cards for DNA storage. In another study, researchers used Whatman FTA^TM^ filter paper to collect onion samples containing infections of pathogenic *Fusarium oxysporum* strains for detection using the PCR method and found that Whatman FTA™ filter paper exhibited potent preservation ability for *Fusarium oxysporum* [37]. In the present study, it was found that Whatman FTA cards exhibited a higher preservation capacity to five types (both laboratory and environmental strains) of bacteria, including *Escherichia coli* O157:H7, *Listeria monocytogenes*, *Salmonella enterica* serovar Enteritidis, and *Staphylococcus aureus* for detection by RT-qPCR. The possible reason that Whatman FTA can store DNA for a longer period of time than conventional methods (e.g., swabs) is that the chemical (e.g., anionic detergents) on the cotton-based cellulose paper has the ability to immobilize genomes of organisms. However, conventional methods do not have this type of chemical and can thus not store DNA for a long period of time [22]. Hence, Whatman FTA cards may be a suitable tool for the routine isolation of foodborne bacteria for molecular diagnosis.

In conclusion, in the present study, it was confirmed that Whatman FTA cards were more suitable for preserving enteric bacteria, such as *Escherichia coli* O157:H7, *Listeria monocytogenes*, *Salmonella enterica* serovar Enteritidis, and *Staphylococcus aureus* than conventional tools, such as swabs when the RT-qPCR method is used to detect bacteria. Therefore, the use of Whatman FTA cards for sample collection and preservation may increase the sensitivity and accuracy for bacterial isolation and diagnosis.

## Figures and Tables

**Table 1:** Primers used in the study.

Bacteria	Gene name		Sequence (5′-3′)	Amplicon size expected (bp)
*Escherichia coli* O157:H7	Shiga toxins (stx1)	Forward	TTTGTYACTGTSACAGCWGAAGCYTTACG	132
		Reverse	CCCCAGTTCARWGTRAGRTCMACRTC	
		Probe-FAM	CTGGATGATCTCAGTGGGCGTTCTTATGTA A	
	Shiga toxins (stx2)	Forward	TTTGTYACTGTSACAGCWGAAGCYTTACG	128
		Reverse	CCCCAGTTCARWGTRAGRTCMACRTC	
		Probe-VIC	TCGTCAGGCACTGTCTGAAACTGCTCC	

*Listeria monocytogenes*	hlyA-177	hlyA-177-F	TGCAAGTCCTAAGACGCCA	120
		hlyA-177-R	CACTGCATCTCCGTGGTATACTAA	
	hlyA-146	hlyA-146-F	AAATCTGTCTCAGGYGATGT	113
		hlyA-146-R	CGATGATTTGAACTTCATCTTTTGC	

*Salmonella enteritidis*	invA	invA-F	CACGCTCTTTCGTCTGGCA	154
		invA-R	TACGGTTCCTTTGACGGTGCGA	

*Staphylococcus aureus*	FemB	FemB-F	AATTAACGAAATGGGCAGAAACA	93
		FemB-R	TGCGCAACACCCTGAACTT	

## Data Availability

All the original data used to support the findings of this study may be released upon application to Tianjin Medical University General Hospital, who can be contacted through Na Yue (yuena19800304@163.com).
